# TME2/342: The Role of the EXtensible Markup Language (XML) for Future Healthcare Application Development

**DOI:** 10.2196/jmir.1.suppl1.e109

**Published:** 1999-09-19

**Authors:** G Noelle, J Dudeck

**Affiliations:** ^1^MED Medicine Online GmbH, Bergisch GladbachGermany

**Keywords:** XML, Standards, Internet, Intranet, Electronic Patient Record

## Abstract

Two years, since the World Wide Web Consortium (W3C) has published the first specification of the eXtensible Markup Language (XML) there exist some concrete tools and applications to work with XML-based data. In particular, new generation Web browsers offer great opportunities to develop new kinds of medical, web-based applications. There are several data-exchange formats in medicine, which have been established in the last years: HL-7, DICOM, EDIFACT and, in the case of Germany, xDT. Whereas communication and information exchange becomes increasingly important, the development of appropriate and necessary interfaces causes problems, rising costs and effort. It has been also recognised that it is difficult to define a standardised interchange format, for one of the major future developments in medical telematics: the electronic patient record (EPR) and its availability on the Internet. Whereas XML, especially in an industrial environment, is celebrated as a generic standard and a solution for all problems concerning e-commerce, in a medical context there are only few applications developed. Nevertheless, the medical environment is an appropriate area for building XML applications: as the information and communication management becomes increasingly important in medical businesses, the role of the Internet changes quickly from an information to a communication medium. The first XML based applications in healthcare show us the advantage for a future engagement of the healthcare industry in XML: such applications are open, easy to extend and cost-effective. Additionally, XML is much more than a simple new data interchange format: many proposals for data query (XQL), data presentation (XSL) and other extensions have been proposed to the W3C and partly realised in medical applications.

